# Seasonal burden of severe influenza virus infection in the critically ill patients, using the Assistance Publique-Hôpitaux de Paris clinical data warehouse: a pilot study

**DOI:** 10.1186/s13613-021-00884-8

**Published:** 2021-07-29

**Authors:** Muriel Fartoukh, Guillaume Voiriot, Laurent Guérin, Jean Damien Ricard, Alain Combes, Morgane Faure, Sarah Benghanem, Etienne de Montmollin, Yacine Tandjaoui-Lambiotte, Antoine Vieillard-Baron, Eric Maury, Jean-Luc Diehl, Keyvan Razazi, Virginie Lemiale, Pierre Trouiller, Benjamin Planquette, Laurent Savale, Nicholas Heming, Jonathan Marey, AP-HP Clinical Data Warehouse, Fabrice Carrat, Nathanael Lapidus, The EPIcuFLU_APHP Group, Michel Djibré, Jean Louis Teboul, Jonathan Messika, Alexandre Demoule, Jean Paul Mira, Jean-François Timsit, Yves Cohen, Bernard Page, Armand Mekontso Dessap, Elie Azoulay, Olivier Sanchez, Marc Humbert, Djillali Annane, Nicolas Roche

**Affiliations:** 1 Service de Médecine Intensive Réanimation, Hôpital Tenon, Assistance Publique-Hôpitaux de Paris, Sorbonne Université, 75020 Paris, France; 2 Groupe de Recherche Clinique CARMAS, Collégium Gallilée, Créteil, France; 3 Service de Médecine Intensive-Réanimation, Hôpital Bicêtre, Assistance Publique-Hôpitaux de Paris, Université Paris-Saclay, Le Kremlin-Bicêtre, France; 4 Medical and Surgical Intensive Care Unit, Assistance Publique-Hôpitaux de Paris, Hôpital Louis Mourier, 92700 Colombes, France; 5 Infection, Antimicrobials, Modelling and Evolution, IAME UMR 1137 Institut National de la Santé et de la Recherche Médicale, Université de Paris, 75018 Paris, France; 6 Service de Médecine Intensive Réanimation, Institut de Cardiologie, Hôpital Pitié-Salpêtrière, Assistance Publique-Hôpitaux de Paris, Sorbonne Université, Paris, France; 7 INSERM-UMRS 1166, iCAN Institute of Cardiometabolism/Nutrition, Sorbonne Université, Paris, France; 8 Service de Médecine Intensive - Réanimation (Département “R3S”), Hôpital Pitié-Salpêtrière, Assistance Publique-Hôpitaux de Paris, Sorbonne Université, Paris, France; 9 UMR_S 1158 Neurophysiologie Respiratoire Expérimentale et Clinique, INSERM, Sorbonne Université, Paris, France; 10 Medical Intensive Care Unit, Groupe Hospitalier Paris Centre-Cochin University Hospital-Assistance Publique-Hôpitaux de Paris, Paris University, 75014 Paris, France; 11 UMR 1137, IAME, Université de Paris, Paris, France; 12 Medical and Infectious Diseases ICU, Bichat-Claude Bernard Hospital, Assistance Publique-Hôpitaux de Paris, Paris, France; 13 Assistance Publique-Hôpitaux de Paris, Service de Réanimation médico-chirurgicale, CHU Avicenne; INSERM U1272 Hypoxie & Poumon, Bobigny, France; 14 Intensive Care Unit, Assistance Publique-Hôpitaux de Paris, University Hospital Ambroise Paré, Boulogne Billancourt, France; 15 Faculty of Medicine Simone Veil, Saint Quentin en Yvelines, France; 16 Inserm U1018, Center for Research in Epidemiology and Population Health (CESP), Faculty of Paris Saclay, Villejuif, France; 17 Service de Réanimation Médicale, Hôpital Saint-Antoine, Assistance Publique-Hôpitaux de Paris, Sorbonne Université, 75571 Paris, France; 18 U 1136, Inserm, Institut Pierre-Louis d’Epidémiologie et de Santé Publique, 75012 Paris, France; 19 Service de Médecine Intensive Réanimation, Hôpital Européen Georges Pompidou, Assistance Publique-Hôpitaux de Paris, Université Paris Descartes, Paris, France; 20 Faculty of Pharmacy, INSERM UMR-S1140, Paris Descartes University, Paris, France; 21 Service de Médecine Intensive Réanimation, Hôpitaux Universitaires Henri Mondor, Assistance Publique-Hôpitaux de Paris, Créteil, France; 22 Groupe de Recherche Clinique CARMAS, Université Paris Est Créteil, Créteil, France; 23 Service de Médecine Intensive et Réanimation, Hôpital Saint-Louis, Assistance Publique-Hôpitaux de Paris, Paris, France; 24 ECSTRA Team, Biostatistics and Clinical Epidemiology, Center of Epidemiology and Biostatistics Sorbonne Paris Cité, Institut National de la Santé et de la Recherche Médicale, Paris Diderot Sorbonne University, Paris, France; 25 Service de Réanimation Polyvalente, Hôpital Antoine Béclère, Assistance Publique-Hôpitaux de Paris, Paris Sud University, Clamart, France; 26 Service de Pneumologie et Soins Intensifs, Hôpital Européen Georges Pompidou, Assistance Publique-Hôpitaux de Paris, Université Paris Descartes, 75015 Paris, France; 27 Biosurgical Research Laboratory (Carpentier Foundation), Assistance Publique-Hôpitaux de Paris, Georges Pompidou European Hospital, 75015 Paris, France; 28 Service de Pneumologie et Soins Intensifs Respiratoires, Hôpital Bicêtre, Assistance Publique-Hôpitaux de Paris, Le Kremlin-Bicêtre, France; 29 Faculty of Medicine, Université Paris-Saclay, Le Kremlin-Bicêtre, France; 30 INSERM UMR_S 999, Le Kremlin-Bicêtre, France; 31 General Intensive Care Unit, Assistance Publique-Hôpitaux de Paris, Raymond-Poincaré Hospital, University of Versailles Saint-Quentin en Yvelines, Garches, France; 32 Service de Pneumologie, Institut Cochin, INSERM U1016, Université de Paris, Assitance Publique-Hôpitaux de Paris, Paris, France; 33 Unité de Santé Publique, INSERM, Institut Pierre Louis d’Epidemiologie et de Sante Publique, Hopital Saint-Antoine, Assistance Publique-Hôpitaux de Paris, Sorbonne Université, 75012 Paris, France

**Keywords:** Epidemic, Influenza, Assistance Publique-Hôpitaux de Paris (AP-HP) clinical data warehouse, Critical care, Prognosis

## Abstract

**Purpose:**

At the critical care level, the flu surveillance system is limited in France, with heterogeneous regional modalities of implementation.

**Materials, patients and methods:**

We aimed at assessing the relevance of the Assistance Publique-Hôpitaux de Paris (AP-HP) clinical data warehouse for estimating the burden of the influenza epidemic on medical adult critical care units of the AP-HP, and outcome of patients during the flu season 2017–2018. This exploratory multi-site epidemiological study comprised all consecutive adult stays (*n* = 320) in 18 medical intensive care units (ICU) or intermediate care wards (ICW) for probable or confirmed *Influenza* virus infection during the 2017–2018 flu season.

**Results:**

Patients admitted to ICU/ICW had low vaccination coverage (21%), required life support in 60% of cases, stayed in the ICU for a median of 8 days, and had high 28-day mortality rate (19.7%; 95% confidence interval 15.5–24.5). Early prognostic factors included age, core temperature, the acute organ failures score, and the early administration of antiviral therapy.

**Conclusions:**

Data directly extracted from the electronic medical records stored in the data warehouse provide detailed clinical, care pathway and prognosis information. The real-time availability should enable to detect and assess the burden of the most severe cases. By a firmer and more acute monitoring and adjustment of care and patient management, hospitals could generate more ICU/ICW capacities, sensitize their emergency department and contribute to the recommendations from health authorities. This pilot study is of particular relevance in the context of emerging epidemics of severe acute respiratory diseases.

**Supplementary Information:**

The online version contains supplementary material available at 10.1186/s13613-021-00884-8.

## Background

Each year, the characteristics of the flu epidemic are likely to evolve [1–5], and may require specific recommendations from the health authorities. In France, the coordination of the epidemiological and virological surveillance has been gradually structured under the auspices of the National Institute for Public Health (Santé Publique France, SPF) (http://invs.santepubliquefrance.fr). At the critical care level, the surveillance system has been developed since the 2009 influenza pandemic, with heterogeneous regional modalities of implementation. In Paris area, individual case report forms of probable or confirmed severe influenza illness are completed by a regional network of 17 sentinel adult medical intensive care units (ICU) and affiliated medical intermediate care wards (ICW) on a voluntary basis, and are sent to SPF every time a new case is diagnosed. Based on those reports, a regional feedback is weekly available, to provide detection and situational awareness regarding the most severe cases in the Paris area.

The relevance of a real-time computerized tool for monitoring and reporting severe cases and their impact on critical care services has been assessed during the H1N1 2009 pandemic in a study involving the REVA research network in connection with the French Intensive Care Society [6], by demonstrating the impact of severe cases on the workload and organization of ICUs. Other efforts have been conducted to improve the assessment of the influenza epidemic severity and its impact on critical care [7, 8]. As part of the implementation of a common clinical information system for all the centers of the Assistance Publique-Hôpitaux de Paris (AP-HP) hospital group, the healthcare data warehouse “Entrepôt de Données de Santé” (EDS AP-HP https://eds.aphp.fr) was set up in 2015 to support non-interventional research and hospital management studies based on data collected during patients’ stays at AP-HP. The EPIcuFLU_APHP research is a pilot multi-site epidemiological study of admissions in adult critical care units (medical ICU/ICW and respiratory ICW) for *Influenza* virus infection. The main objective was to assess the burden of the epidemic on critical care units, by describing the severity and outcomes of adult patients admitted to the ICUs/ICWs of the APHP network during the influenza season. The primary and secondary endpoints were the in-hospital mortality within 28 days of ICU/ICW admission with a diagnosis of influenza infection; ICU/ICW and hospital lengths of stay and in-hospital mortality rates, and the early prognostic factors associated with 28-day mortality, based on data available during the first 24 h of ICU/ICW admission.

## Materials and methods

### Study design and population

The research was conducted during the 2017–2018 influenza epidemic in France [9], from November 1st 2017 to May 31 2018, in the medical adult ICUs/ICWs and respiratory ICWs of the AP-HP, Paris, France. The 18 participating centers (15 medical ICUs and affiliated ICWs, and three respiratory ICWs) are listed in Additional file 1: Table S1. All patients with severe *Influenza* virus infection consecutively admitted to the participating centers during the 2017–2018 influenza season were identified using the medical information system coding database (Programme de Médicalisation des Systèmes d’Information [PMSI]). The selection of adult stays (15 years and over) was performed on Diagnosis Related Groups in ICU/ICW, with the mention of “*Influenza*” in one of the coded diagnoses, using the International Classification of Diseases ICD-10 diagnosis codes (see Additional file 1). The diagnosis of *Influenza* virus infection was definite or probable, whether it was eventually microbiologically confirmed or not. Inter-institutional transfers within the AP-HP centers were considered in the patient care pathway by grouping together patient stays to obtain a database of unique patients.

### Data recorded

For each selected case, baseline demographics and comorbidities, initial clinical presentation and vital signs, therapeutic management, ICU and hospital lengths of stay and vital status at discharge were extracted from the electronic health records (see Additional file 1).

### Statistics

The characteristics of the population are described and compared according to their vital status at day 28 (D28). Qualitative variables are described by their frequencies and percentages of observed values, quantitative variables by their medians and interquartile ranges (IQR). Variables associated with 28-day mortality were identified using univariable Cox regression, with follow-up censored on D28. Hazard ratios (HR) are reported with their 95% confidence interval (CI). A sensitivity analysis was conducted to account for the multicenter design with the use of frailty models. Multivariable models were built to identify factors independently associated with 28-day mortality. Variables were included in the multivariable analysis from a practical perspective, when the information they provided was deemed clinically relevant and easily available on admission: two models were built from age > 65 years, comorbid conditions, abnormal core temperature (less than 35 °C or at least 40 °C), antiviral treatment on admission and a severity score [either the acute organ failure score (SOFA) or the CURB65 score]. Age > 65 years was removed from the model with the CURB65 score, as it already was a component of this score. When values were missing for components needed to compute the PSI or CURB65 scores [10, 11], multiple imputation of these variables was used to compute these scores for all patients. Individual imputed patient’s scores were averaged and rounded over 30 imputed datasets. Regression results relying on these scores were obtained by applying Rubin’s rule on these datasets. All tests were two-tailed and *p* values < 0.05 were considered significant. Statistical analysis was conducted with R version 3.6.3 (R Core Team 2019; R foundation for statistical Computing, Vienna, Austria).

### Ethical considerations

The EPIcuFLU_APHP research is a multicenter non-interventional data-based research using the care data collected during patients’ stays at AP-HP. It was approved by the Scientific and Ethics Committee of the EDS AP-HP, which was authorized by the National Commission on Informatics and Liberty (CNIL) for such a non-interventional data-based research with no informed consent. There is no processing of indirectly identifiable data, or chaining with data from other sources, or long-term patient follow-up for this research. The access to data from different units and services of the AP-HP was the subject of the requesting investigator’s fair information to data-producing professionals including the department heads or their representatives to ensure that they did not object to the use of patient’s data they had taken care of.

## Results

**Fig. 1:**
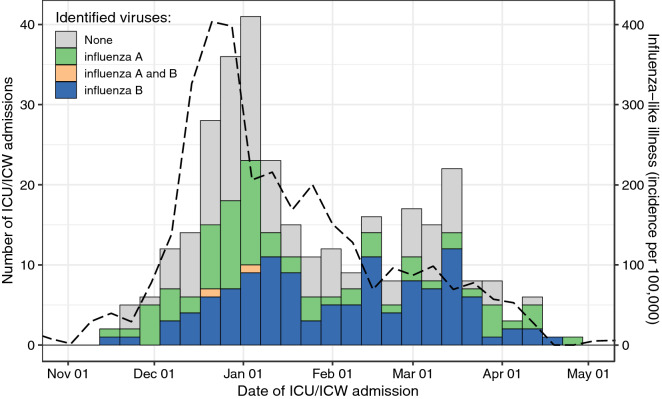
ICU/ICW admissions throughout the 2017–2018 influenza season according to virus type. ICU/ICW weekly admissions in ICU/ICW, according to identified influenza virus types (bar plot, left axis) and incidence of influenza-like illness in Paris area (dashed line, right axis) [reference: https://www.sentiweb.fr/]

During the study period, 320 patients with probable or confirmed influenza infection were admitted to the ICU/ICW of the participating centers, a median of 3 [1–5] days after symptoms onset, and 76 patients (24%) had already started taking oseltamivir before ICU/ICW admission (Table 1). The patients (188 men; 58.8%) were aged 63.2 [52.3–73.4] years, had moderate overweight (body mass index > 30, *n* = 29; 9.1%) and often comorbid conditions, mainly congestive heart failure (*n* = 54; 16.9%), chronic renal disease (*n* = 53; 16.6%), and neoplastic disease (*n* = 42; 13.1%). Most patients had acute respiratory failure (*n* = 211; 65.9%) on ICU/ICW admission. The SAPSII score and SOFA score were 37 [28–55] and 5 [2–8], respectively. About 13% and 3% of the data were missing for the variables needed to compute the PSI and CURB65 scores, respectively. Most patients were in the highest PSI risk classes (PSI IV–V: *n* = 262; 81.9%), and 146 patients (45.7%) had a CURB65 higher than 2 (Table 2). At least one factor targeted by the vaccination recommendations (Additional file 1: Table S2) was identified in 270 patients (84.4%), but only 52/245 patients (21.2%) in whom this information was reported had been vaccinated. Diagnostic PCR tests were mainly performed on nasopharyngeal swabs or nasopharyngeal aspirates (80%). Influenza infection was laboratory-confirmed in 196 patients (61.3%). The viruses A (H1N1) pdm09 and B/Yamagata were the main circulating viruses (Table 3, Fig. 1).

**Table 1:** Characteristics of the population on ICU/ICW admission

Variable	All participants *n* = 320	Survivors *n* = 257	Non-survivors *n* = 63
**Age (years), median [IQR]**	63.2 [52.30–73.4]	62.6 [50.3–71.3]	69.0 [60.0–81.0]
**Age > 65 years**	147 (45.6)	110 (42.8)	37 (58.7)
**Sex (male), n (%)**	188 (58.8)	148 (57.6)	40 (63.5)
**Obesity**^a^**, n (%)**	29 (9.1)	23 (8.9)	6 (9.5)
**Comorbid conditions, n (%)**	130 (40.6)	99 (38.6)	31 (49.2)
Neoplastic disease	42 (13.1)	30 (11.7)	12 (19.0)
Congestive heart failure	54 (16.9)	43 (16.7)	11 (17.5)
Cerebrovascular disease	16 (5)	11 (4.3)	5 (7.9)
Renal disease	53 (16.6)	39 (15.2)	14 (22.2)
Liver disease	5 (1.6)	2 (0.8)	3 (4.8)
**Influenza vaccination**^**b**^, **n (%)**	52 (21.2)	43 (21.5)	9 (20.0)
**At least one factor targeted by the vaccination **^c^**, n (%)**	270 (84.4)	212 (82.5)	58 (92.1)
**Origin, n (%)**			
Emergency department	153 (47.8)	121 (47.1)	32 (50.8)
Other hospital wards	22 (6.9)	18 (7.0)	4 (6.3)
Nursing home residence	11 (3.4)	9 (3.5)	2 (3.2)
Out-of-hospital emergency services	134 (41.9)	109 (42.4)	25 (39.7)
Healthcare worker	2 (0.6)	1 (0.4)	1 (1.6)
**Information before ICU/ICW admission, n (%)**			
Time from symptoms onset to ICU/ICW referral (days), median [IQR]	3 [1–5]	3 [1–5]	3 [1–6]
Treatments before admission, *n* (%)	220 (69.4)	179 (70.2)	41 (67.2)
Antimicrobial drugs	196 (61.3)	160 (62.3)	36 (57.1)
Antibiotics	179 (56.5)	146 (57.3)	33 (53.2)
Antiviral therapy (oseltamivir)	76 (24.0)	64 (25.1)	12 (19.4)
Anti-inflammatory drugs	53 (16.6)	44 (17.1)	9 (14.3)
Steroids	39 (12.3)	33 (12.9)	6 (9.7)
NSAIDs	17 (5.4)	13 (5.1)	4 (6.5)
**Clinical features during the first 24 h of ICU/ICW admission, n (%)**			
Altered mental status (CGS < 14)	83 (28.2)	66 (27.8)	17 (29.8)
Pulse ≥ 125 per minute	92 (28.8)	72 (27.9)	20 (32.4)
Respiratory rate ≥ 30 per minute or MV	211 (65.9)	165 (64.0)	46 (73.8)
Systolic blood pressure < 90 mm Hg or diastolic blood pressure < 60 mm Hg or vasopressors	116 (36.2)	81 (31.3)	35 (56.0)
Temperature < 35 °C or ≥ 40 °C	41 (13.0)	23 (9.0)	18 (29.5)
**Radiographic findings during the first 24 h of ICU/ICW admission, n (%)**			
Bilateral infiltrate	200 (62.5)	153 (59.5)	47 (74.6)
Pleural effusion	42 (13.1)	30 (11.7)	12 (19.0)

Percentages were calculated after removing observations with missing data

*CGS* Coma Glasgow scale, *ICU* intensive care unit, *ICW* intermediate care wards, *MV* mechanical ventilation, *NSAIDs* non-steroidal anti-inflammatory drugs

^a^Obesity defined as Body Mass Index (kg/m^2^) > 30 kg/m^2^

^b^History of influenza vaccination was the variable with the largest amount of missing data; the reported percentages are 43/(257 − 57) = 21.5%, 9/(63 − 18) = 20.0% and 52/(320 − 75) = 21.2%

^c^Pregnancy, obesity (BMI > 30), 65-year old subjects and over, nursing home residency regardless of age, type 1 and 2 diabetes, chronic respiratory disease (chronic broncho-pulmonary diseases including asthma, broncho-pulmonary dysplasia and cystic fibrosis, chronic respiratory insufficiency), cardiac disease (congenital heart disease, heart failure, valvular disease, severe arrhythmia, coronary disease), neurological or muscle disease (stroke, severe forms of neurological and muscular disorders, para and tetraplegia with diaphragmatic involvement), renal disease (severe chronic renal insufficiency, nephrotic syndrome), immunosuppressive state (primary or acquired immune deficiency, except regular treatment with immunoglobulins, HIV infection and AIDS, solid transplantation), and others (hepatopathy, sickle cell disease, healthcare professionals) (see Additional file 1: Table S2)

**Table 2:** Severity of the disease during the first 24 hours of ICU/ICW admission

Variable	All participants *n* = 320	Survivors *n* = 257	Non-survivors *n* = 63
**Acute organ failure**			
Respiratory failure			
RR > 30/min or mechanical ventilation, *n* (%)	211 (65.9)	165 (64.0)	46 (73.8)
PaO_2_/FiO_2_ ratio (mmHg), median [IQR]	187 [90–336]	200 [98–350]	142 [73–260]
ARDS, *n* (%)	120 (37.7)	82 (32.0)	38 (61.0)
Shock			
SBP < 90, DBP < 60 or vasopressors, *n* (%)	116 (36.2)	81 (31.3)	35 (56.0)
Renal failure			
Renal replacement therapy, *n* (%)	22 (6.9)	12 (4.7)	10 (15.9)
Altered mental status (CGS < 14), *n* (%)	83 (28.2)	66 (27.8)	17 (29.8)
**Severity scores**			
SAPS II (points), median [IQR]	37 [28–55]	34 [26–48]	59 [40–81]
SOFA (points), median [IQR]	5 [2–8]	4 [2–7]	8 [4–14]
Pneumonia Severity Index, median [IQR]	129 [100–160]	122 [96–153]	152 [131–186]
Risk class, n (%)			
II	35 (7.9)	25 (9.7)	0 (0.0)
III	33 (10.2)	31 (11.9)	2 (3.3)
IV	105 (32.8)	92 (35.7)	13 (21.4)
V	157 (49.1)	110 (42.8)	47 (75.2)
CURB65, median [IQR]	2 [1–3]	2 [1–3]	3 [2–4]
Risk class, *n* (%)			
0	24 (7.4)	21 (8.2)	3 (4.1)
1	67 (21.0)	58 (22.6)	9 (14.5)
2	83 (26.0)	72 (28.1)	11 (17.2)
3	86 (26.7)	69 (26.8)	17 (26.3)
4	41 (12.9)	26 (10.0)	15 (15.4)
5	19 (6.1)	11 (4.3)	8 (8.4)

Components of the severity scores were imputed when missing: RR > 30/min or mechanical ventilation (*n* = 6), PaO_2_/FiO_2_ ratio (*n* = 96), ARDS (*n* = 9), shock (*n* = 6), altered mental status (*n* = 26), SAPS II (*n* = 6). Patient counts for these scores were rounded over the 30 imputed datasets

*RR* respiratory rate, *PaO*_*2*_ partial pressure of oxygen, *FiO*_*2*_ fraction of inspired oxygen, *ARDS* acute respiratory distress syndrome, *SBP/DBP* systolic/diastolic blood pressure, *CGS* Glasgow coma scale, *SAPS* simplified acute physiology score, *SOFA* sequential organ failure assessment

**Table 3:** Microbiological diagnosis

Variable	All participants *n* = 320	Survivors *n* = 257	Non-survivors *n* = 63
Virus sampling			
Nasopharyngeal swab	249 (77.8)	203 (79.0)	46 (73.0)
Nasopharyngeal aspirate	10 (3.1)	7 (2.7)	3 (4.8)
Sputum	14 (4.4)	10 (3.9)	4 (6.3)
Bronchial aspirate	31 (9.7)	23 (8.9)	8 (12.7)

Influenza virus type and subtype identified in patients with at least one positive test		*n* = 196	*n* = 155	*n* = 41
Virus A^a^		78 (39.8)	59 (38.1)	19 (46.3)
A/H3N2^a^		10 (5.1)	6 (3.9)	4 (9.8)
A/H1N1pdm^a^		68 (34.7)	53 (32.2)	15 (36.6)
Virus B^a^		120 (61.2)	96 (61.9)	24 (58.6)

^a^Positive tests are reported as counts and percentages in patients with at least one positive test

Altogether, vital support was required in up to 60% of critically ill patients during ICU/ICW stay, including mechanical ventilation (*n* = 182; 56.9%), vasopressors (*n* = 100; 31.2%), and renal replacement therapy (*n* = 39; 12.2%) (Table 4). Additional therapies were administered in the most severe patients, including oseltamivir (*n* = 278; 87%), steroids (*n* = 67; 20.9%), inhaled nitric oxide (NO, *n* = 17; 5.3%) and extracorporeal membrane oxygenation (ECMO, *n* = 6; 1.9%). Lengths of ICU and hospital stay were 8 [4–15] days and 15 [8–28] days, respectively.

**Table 4:** Treatments administered in ICU and outcomes

Variable	All participants *n* = 320	Survivors *n* = 257	Non-survivors *n* = 63
**Organ support and additional therapies, n (%)**			
Mechanical ventilation^a^	182 (56.9)	136 (52.9)	46 (73.0)
Invasive mechanical ventilation	116 (36.2)	77 (30.0)	39 (61.9)
Neuromuscular blocking agents	17 (5.3)	13 (5.1)	4 (6.3)
Prone positioning	35 (10.9)	23 (8.9)	12 (19.0)
NO	17 (5.3)	13 (5.1)	4 (6.3)
ECMO	6 (1.9)	3 (1.2)	3 (4.8)
Vasopressors	100 (31.2)	62 (24.1)	38 (60.3)
Renal replacement therapy	39 (12.2)	22 (8.6)	17 (27)
Steroids^b^	67 (20.9)	52 (20.2)	15 (23.8)
Oseltamivir^c^	278 (86.9)	232 (90.3)	46 (73.0)
**Length of stay (days), median [IQR]**			
ICU	8 [4–15]	8 [4–15]	7 [2–15.5]
Hospital	15 [8–28]	16 [9–31]	10 [3.5–18.5]

*NO* nitric oxide, *ECMO* extracorporeal membrane oxygenation

^a^Invasive mechanical ventilation with endotracheal intubation, non-invasive positive pressure ventilation and high flow nasal oxygen therapies were included in the category mechanical ventilation

^b^Steroids included hydrocortisone and methylprednisolone

^c^Oseltamivir was either continued (*n* = 76) or introduced within the first 24 h of ICU/ICW referral (*n* = 202)

**Fig. 2:**
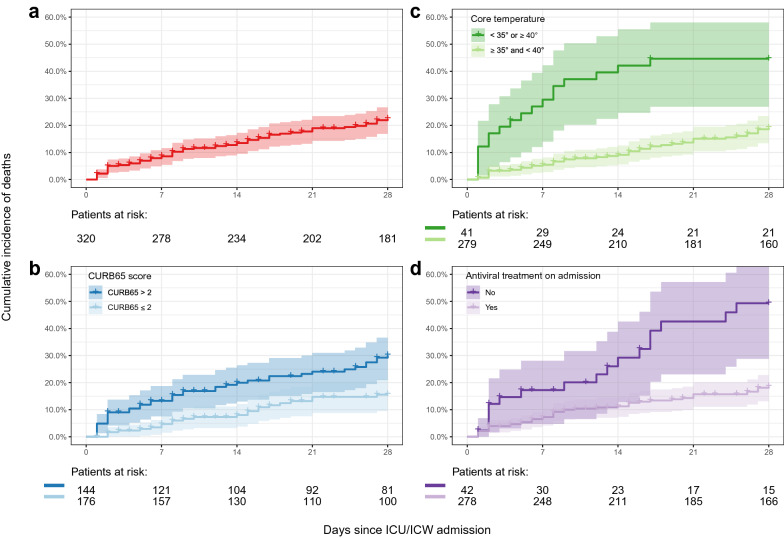
Cumulative incidence of deaths following ICU/ICW admission, overall (**a**), and according to the CURB65 score (**b**), core temperature (**c**) and antiviral treatment on admission (**d**). Panels illustrate the combination of the independant factors associated with 28-day mortality (second model), including the class of CURB65 (which may be either > 2 or ≤ 2; panel **a**), the core temperature on ICU admission (which may be either < 35 °C or ≥ 40 °C, or ≥ 35 °C and < 40 °C; panel **c**), and the antiviral treatment administered on ICU admission (panel **d**)

At D28, 63 patients (19.7%) had died, 33 (10.3%) were still hospitalized in the ICU, while 224 (70.0%) had been discharged to conventional wards or long-term rehabilitation care units. The distribution of ICU admissions relative to the incidence of influenza-like illness in the Paris area is shown Fig. 1. Variables associated with 28-day-mortality are listed in Table 5, with hazard ratios for the risk of death 28 days after ICU/ICW admission. Two multivariable models were built, attempting at providing pragmatic and easily available information for clinical routine use. The first model identified four factors available during the first 24 h of ICU/ICW admission and independently associated with 28-day mortality: age > 65 years (HR: 1.79, 95% CI 1.02–3.16 *p* = 0.043), core temperature < 35 °C or ≥ 40 °C (HR: 3.06, 95% CI 1.73–5.42; *p* < 0.001), acute organ failure score (HR: 1.16, 95% CI 1.10–1.23 per 1-point increase; *p* < 0.001), and antiviral treatment on admission (HR: 0.45, 95% CI 0.24–0.85; *p* = 0.014). The scores dedicated to pneumonia (PSI and CURB65 scores) were not entered into that first model. The second model identified the CURB65 score > 2 (HR: 1.30, 95% CI 1.06–1.60; *p* = 0.014), core temperature < 35 °C or ≥ 40 °C (HR: 2.94, 95% CI 1.67–5.19; *p* < 0.001), and antiviral treatment on admission (HR: 0.34, 95% CI 0.19–0.61; *p* < 0.001) as being independently associated with 28-day mortality. Both these models were adjusted on the presence of comorbid conditions, which was not associated with mortality. The cumulative incidence of deaths over the 28-day follow-up is shown in Fig. 2, overall (A) and according to the CURB65 score (B), abnormal core temperature (C) and antiviral treatment on ICU admission (D). Frailty models accounting for the multicenter design yielded similar results (not reported).

**Table 5:** Hazard ratios for the risk of death up to day 28 after admission to ICU/ICW

Variable	Univariable analysis		Multivariable analysis	
	HR (95% CI)	*p*	First model HR (95% CI)	Second model HR (95% CI)
**Age (per 10 years)**	1.42 (1.18–1.71)	0.0002	–	–
**Age > 65 years**	1.70 (1.03–2.81)	0.039	1.79 (1.02–3.16)*	–
**Sex (male)**	1.20 (0.72–2.00)	0.49	–	–
**Obesity**	1.11 (0.48–2.58)	0.81	–	–
**Comorbid conditions**	1.33 (0.81–2.18)	0.26	1.16 (0.69–1.93)	1.17 (0.70–1.97)
Neoplastic disease	1.50 (0.80–2.82)	0.20	–	–
Congestive heart failure	1.00 (0.52–1.92)	0.99	–	–
Cerebrovascular disease	1.78 (0.72–4.45)	0.21	–	–
Renal disease	1.36 (0.75–2.46)	0.31	–	–
Liver disease	6.64 (2.05–21.54)	0.0016	–	–
**Status of influenza vaccination**	1.03 (0.48–2.21)	0.94	–	–
**At least one factor targeted by the vaccination**^a^	1.87 (0.75–4.67)	0.18	–	–
**Time to referral from symptoms onset (per day)**	1.00 (0.97–1.03)	0.99	–	–
**Treatments before admission**	0.89 (0.52–1.52)	0.67		
Antimicrobial drugs	0.81 (0.49–1.34)	0.41	–	–
Antibiotics	0.85 (0.51–1.39)	0.51	–	–
Antiviral therapy (oseltamivir)	0.70 (0.37–1.31)	0.26	–	–
Anti-inflammatory drugs	0.89 (0.44–1.8)	0.75	–	–
Steroids	0.74 (0.32–1.7)	0.47	–	–
NSAIDs	1.62 (0.59–4.41)	0.35	–	–
**Physical exam during the first 24 h of ICU/ICW admission**				
Pulse ≥ 125 per minute	1.21 (0.71–2.06)	0.48	–	–
Temperature < 35 °C or ≥ 40 °C	3.37 (1.96–5.81)	0.00002	3.06 (1.73–5.42)**	2.94 (1.67–5.19)**
Respiratory rate ≥ 30 per minute or MV	1.49 (0.85–2.62)	0.17	–	–
Systolic blood pressure < 90 mm Hg or diastolic blood pressure < 60 mm Hg or vasopressors	2.4 (1.44–3.99)	0.0007	–	–
Renal replacement therapy	2.6 (1.32–5.11)	0.0056	–	–
Altered mental status (CGS < 14)	1.05 (0.6–1.84)	0.87	–	–
**Initial severity (scores)**				
SAPS2 (points)	1.04 (1.03–1.05)	< 10*e*−5	–	–
SOFA (points)	1.15 (1.09–1.21)	< 10*e*−5	1.16 (1.10–1.23)**	–
Pneumonia Severity Index, Class IV–V	7.30 (1.04–51.31)	0.046	–	–
CURB65 > 2	2.20 (1.30–3.75)	0.0035	–	1.30 (1.06–1.60)*
**Antiviral therapy**				
Oseltamivir (ongoing or introduced after ICU admission)	0.32 (0.18–0.56)	0.00017	0.45 (0.24–0.85)*	0.34 (0.19–0.61)**

^*^p < 0.05; **p < 0.001

^a^Pregnancy, obesity (body mass index > 30 kg/m^2^), 65-year-old subjects and over, nursing home residency regardless of age, type 1 and 2 diabetes, chronic respiratory disease (chronic broncho-pulmonary diseases including asthma, broncho-pulmonary dysplasia and cystic fibrosis, chronic respiratory insufficiency), cardiac disease (congenital heart disease, heart failure, valvular disease, severe arrhythmia, coronary disease), neurological or muscle disease (stroke, severe forms of neurological and muscular disorders, para and tetraplegia with diaphragmatic involvement), renal disease (severe chronic renal insufficiency, nephrotic syndrome), immunosuppressive status (primary or acquired immune deficiency, except regular treatment with immunoglobulins, HIV infection and AIDS, solid transplantation), and others (liver disease, sickle cell disease, healthcare professionals). See Additional file 1: Table S2

## Discussion

This non-interventional research was conducted during the flu season 2017–2018 in the adult ICUs/ICWs network of the AP-HP to assess the burden of the epidemic in critically ill patients, using the healthcare data warehouse of the Paris university hospitals network (EDS AP-HP). Our findings highlight the high burden and severity of influenza on critical care services, involving patients with low vaccination coverage, requiring life support in 60% of cases, and having prolonged length of stay and a high (20%) mortality rate.

Early prognostic factors were mainly related to age, fever, the severity of the acute infection, as well as to the early administration of antiviral treatment. As expected, both the generic severity scores and scores dedicated to community-acquired pneumonia were strongly associated with 28-day mortality. The two final selected models highlight the importance of age and acute organ failure, two prognostic elements readily available at initial evaluation of patients. On the basis of the 2016–2017 flu season in France, we had estimated that approximately 300 patients would be hospitalized in the participating adult ICUs over a similar 2017–2018 season, i.e., with a “severe” profile, and that the expected number of deaths at 28 days would range between 43 (moderate epidemic) and 86 (severe epidemic). Altogether, we recorded 320 ICU stays involving 65-year or older and fragile patients with comorbid conditions, and 73 (22.8%) in-hospital deaths. At least one factor targeted by the vaccination recommendations was identified in most of them, but only 20% had received vaccination, reinforcing the fact that efforts to foster preventive strategies including vaccination are needed. Most patients were directly referred to the ICUs from out-of-hospital emergency services or EDs, about 3 days after symptoms onset. The patients were receiving antimicrobial therapies (antibiotics and/or oseltamivir) and/or anti-inflammatory drugs (steroids and/or NSAIDs) before ICU referral in 61.3% and 16.6% of cases, respectively. The initial severity was high, as demonstrated by the presence of an acute organ failure in two-thirds of the cases, involving mainly the lungs. The generic scores [12, 13] as well as the scores dedicated to community-acquired pneumonia [10, 11] were concordant with that severity, suggesting an overall probability of death ranging from 20 to 30%. The observed 28-day mortality was 19.7% (95% CI 15.5–24.5). These findings are in accordance with different sources of data collected in France for the 2017–2018 season [14]. These surveillance data showed that the epidemic started early, had a significant severity, and was exceptionally long in the context of insufficient vaccination coverage in France, and suboptimal vaccine efficacy. The atypical dynamics was related to the successive circulation of the A (H1N1) pdm09 and B/Yamagata viruses and may have contributed to those findings [14, 15]. Antiviral therapy should be initiated as early as possible, the earlier initiation being more likely to provide benefit. However, randomized controlled trial data are not available to assess the impact of oseltamivir use among hospitalized patients with severe disease [16]. When replacing oseltamivir on ICU/ICW admission by oseltamivir started before ICU/ICW admission in the selected multivariable models, the hazard ratios for death within 28 days were still in favor of a lower mortality, yet the difference was not significant (HR: 0.65 [95% CI 0.34–1.24] and 0.62 [0.32–1.19] for the models with age > 65 years and with the CURB65 score, respectively). These results suggest that even though antiviral treatment is certainly beneficial for these patients when taken before ICU/ICW admission (which our study was not designed to evaluate), its benefit remains likely even when taken early on ICU/ICW admission. Thus, our findings confirm the high impact of the disease for the population at risk and strengthen the need for prevention, especially by promoting higher vaccine coverage among people at risk and compliance to control measures to limit the spread of the virus; our study also strongly suggest the potential benefit of early antiviral treatment for subjects at risk.

### Limitations

The limitations of our clinical research project are related to the fact that it was one of the very first designed to use the Assistance Publique-Hôpitaux de Paris clinical data warehouse. Research on health data warehouses has become increasingly popular in the last few years and such databases may be considered as a major source of information for clinical research in a near future. This opportunity obviously relies on the possibility to effectively extract meaningful information from this massive amount of data that were collected without a predefined research question or even a research objective. This paradigm is far from the traditional clinical research settings that are mostly used to answer such questions, and there is still a lot to do to transform these databases into the powerful research tools they may become. In our study, some information were lacking, such as the diagnosis of early bacterial co-infection or the do-not-resuscitate order decisions that may have had an impact on the outcome of patients. The reproducibility and the validity of the results provided by the Assistance Publique-Hôpitaux de Paris clinical data warehouse are a major concern, and should be evaluated in different fields, for instance by comparing them with the results obtained from “gold-standard” dedicated studies relative to a specific research question. Since we started this study, a lot has been made to enhance the opportunity to use the Assistance Publique-Hôpitaux de Paris clinical data warehouse for research, and new challenges are constantly addressed so that this database may be part of the clinical researcher’s toolbox within a few years. New healthcare data types are continuously integrated into the warehouse, which now contains data on ICD-10 diagnosis codes, medical procedures, laboratory results including microbiology, imaging, medication dispensing and medical documents regarding over 10 million patients in 39 hospitals.

To summarize, the main difficulties of this pilot work were linked to the fact that the healthcare data warehouse was not designed a priori for real-time monitoring. However, coding of diagnoses and medical procedures over time in critical care units and regular updates and extractions every 24 or 48 h are possible and could help achieve this goal. Data directly extracted from electronic medical records provide very useful and detailed clinical and care pathway information with more precision than those of the current regional surveillance network. These data should be available in a timely fashion with the aim of providing situational awareness regarding the most severe cases and thereby improving their detection. In the era of emerging infectious diseases and pandemics, the development of tools to monitor easily and in real time the progression of an epidemic, its severity and its impact on critical care organizations is a public healthcare imperative [17–20]. Our pilot study is of particular relevance in the context of emerging respiratory viral pandemics, such as the COVID19 pandemic. It supports the usefulness of institutional tools to monitor the burden of the most severe cases in real time and to inform critical care services and health authorities, to adapt the healthcare system in a timely fashion by generating more ICU/ICW capacities, sensitizing the emergency departments and finally contributing to the recommendations from health authorities.

## Supplementary Information

**Additional file 1.** Selection of the population and Data recorded. **Table S1.** Participating centres. **Table S2.** Factor(s) targeted by the vaccination in the studied population.

## Data Availability

The dataset used and analyzed during the current study are available from the AP-HP Clinical Data Warehouse on reasonable request.

## Abbreviations

AP-HP: Assistance Publique-Hôpitaux de Paris
ARDS: Acute respiratory distress syndrome
DBP: Diastolic blood pressure
ECMO: Extracorporeal membrane oxygenation
CGS: Glasgow coma scale
ICU: Intensive care unit
ICW: Intermediate care ward
NO: Nitric oxide
RR: Respiratory rate
PaO_2_: Partial pressure of oxygen
FiO_2_: Fraction of inspired oxygen
SAPS: Simplified acute physiology score
SBP: Systolic blood pressure
SOFA: Sequential organ failure assessment
