# Supplementary material for: Seasonal burden of severe influenza virus infection in the critically ill patients, using the Assistance Publique-Hôpitaux de Paris clinical data warehouse: a pilot study
Source: Ann Intensive Care. 2021 Jul 29;11:117. doi: 10.1186/s13613-021-00884-8 (PMC8319596; doi:10.1186/s13613-021-00884-8)
Supplement: Supplementary file 1 — Additional file 1. Selection of the population and Data recorded. Table S1. Participating centres. Table S2. Factor(s) targeted by the vaccination in the studied population. [file 13613_2021_884_MOESM1_ESM.doc]

**Seasonal burden of severe influenza virus infection in the critically ill patients, using the Assistance Publique - Hôpitaux de Paris Clinical Data Warehouse: a pilot study.**

***Additional file 1***

**Patients and methods**

**Selection of the population**

The fifteen participating ICUs and three respiratory ICWs are detailed **Table E1**. All consecutive patients with severe *Influenza* virus infection admitted to the participating centers were identified using the medical information system coding database (Programme de Médicalisation des Systèmes d’Information [PMSI]. The selection of adult stays (15 years and over) was performed on Diagnosis Related Groups in ICU/ICW, with the mention of "*Influenza*" in one of the coded diagnoses, using the following International Classification of Diseases ICD-10 diagnosis codes: J09, J100, J101, J108, J110, J111, J118, J129, I41.1* and G05.1*.

**Data recorded**

For each selected case, baseline demographics, initial clinical presentation and vital signs, therapeutic management, ICU and hospital lengths of stay and vital status at discharge were recorded.

***Demographics*** included age, sex, weight, height, status of influenza vaccination for the current epidemic, and comorbid conditions or associated pathologies as individualized in the PSI (12) (neoplastic disease, congestive heart failure, cerebrovascular disease, renal disease, and liver disease).

***Risk factors*** targeted by the vaccination included the following (**Table E2**): pregnancy, obesity (BMI>30), age 65-year or older, nursing home residency regardless of age, type 1 and 2 diabetes, chronic respiratory disease (chronic broncho-pulmonary diseases including asthma, broncho-pulmonary dysplasia and cystic fibrosis, chronic respiratory insufficiency), cardiac disease (congenital heart disease, heart failure, valvular disease, severe arrhythmia, coronary disease), neurological or muscle disease (stroke, severe forms of neurological and muscular disorders, para and tetraplegia with diaphragmatic involvement), renal disease (severe chronic renal insufficiency, nephrotic syndrome), immunosuppressive state (primary or acquired immune deficiency, except regular treatment with immunoglobulins, HIV infection and AIDS, solid transplantation), and others (hepatopathy, sickle cell disease, health-care professionals).

The ***care pathway*** included direct ICU admission, transfer from the ED or conventional ward and time to transfer, lengths of ICU and hospital stay and vital status at discharge.

The ***therapeutic management*** detailed (i) treatments administered on admission (during the first 24 hours) and during ICU stay: antiviral treatment and adjuvant treatments such as steroids and antibiotics; (ii) vital organ support therapies on admission (during the first 24 hours) and during ICU stay for respiratory failure (high-flow oxygen, mechanical ventilation, extracorporeal membrane oxygenation (ECMO), nitric oxide (NO), neuromuscular blocking agents, prone position, renal failure (renal replacement therapy) and shock (vasopressors).

The following ***biological variables*** were recorded on admission (during the first 24 hours): creatinine, urea; pH, PaO2, PaO2/FiO2 ratio (if applicable); platelets, leukocytes, and lymphocytes counts, hematocrit; bilirubin, natremia, blood glucose, albumin, and procalcitonin.

The ***initial severity*** of influenza infection on admission (during the first 24 hours) was assessed by generic scores (SAPS2 (11) and SOFA (10)), and by the specific scores for community acquired pneumonia (i.e., PSI (12) and CURB65 (13)). Acute organ failure was defined according to the SOFA score (10). Acute respiratory distress syndrome (ARDS) was defined according to the Berlin definition (21).

**Table S1**. Participating centres

| AP-HP, Hospital | University | Unit | No of patients |
| --- | --- | --- | --- |
| Tenon | Sorbonne Université | Service de Médecine intensive réanimation | 28 |
| Saint Antoine |  | Service de Médecine intensive réanimation | 18 |
| Pitié Salpêtrière |  | Service de Médecine intensive réanimation, Département "R3S" | 26 |
| Pitié Salpêtrière |  | Service de Médecine intensive réanimation | 27 |
| Saint-Louis | Université de Paris | Service de Médecine intensive réanimation | 16 |
| Bichat |  | Service de Médecine intensive réanimation et Maladies infectieuses | 21 |
| Louis Mourier |  | Service de Médecine intensive réanimation | 34 |
| Cochin | Paris Centre | Service de Médecine intensive réanimation | 26 |
| Cochin |  | Unité de soins intensifs de Pneumologie | 3 |
| Hôpital Européen  Georges-Pompidou |  | Unité de soins intensifs de Pneumologie | 14 |
| Hôpital Européen  Georges-Pompidou |  | Service de Médecine intensive réanimation | 17 |
| Henri Mondor | Université Paris Est Créteil | Service de Médecine intensive réanimation | 16 |
| Avicenne | Paris-Seine-Saint-Denis | Réanimation médicochirurgicale | 20 |
| Le Kremlin-Bicêtre | Université Paris Saclay | Service de Médecine intensive réanimation | 39 |
| Le Kremlin-Bicêtre |  | Unité de soins intensifs de Pneumologie | 6 |
| Antoine Béclère |  | Réanimation polyvalente | 15 |
| Ambroise Paré | Université Versailles  Saint-Quentin en Yvelines | Réanimation polyvalente | 19 |
| Raymond Poincaré |  | Réanimation polyvalente | 6 |

**Table S2**. Factor(s) targeted by the vaccination in the studied population

| **Factor targeted by the vaccination*,**  **n (%)** | **All participants**  **n=320** | **Survivors**  **n=257** | **Non-survivors**  **n=63** |
| --- | --- | --- | --- |
| Pregnancy | 3 (0.9) | 3 (1.2) | 0 (0.0) |
| Obesity (BMI>30) | 29 (9.1) | 23 (8.9) | 6 (9.5) |
| age 65-year or older | 147 (45.9) | 110 (42.8) | 37 (58.7) |
| Nursing home | 22 (6.9) | 16 (6.2) | 6 (9.5) |
| Type 1 and 2 diabetes | 79 (24.7) | 63 (24.5) | 16 (25.4) |
| Chronic respiratory disease | 106 (33.1) | 83 (32.3) | 23 (36.5) |
| Cardiac disease | 77 (24.1) | 56 (21.8) | 21 (33.3) |
| Neurological or muscle disease | 22 (6.9) | 18 (7) | 4 (6.3) |
| Renal disease | 53 (16.6) | 39 (15.2) | 14 (22.2) |
| Immunosuppressive status | 77 (24.1) | 56 (21.8) | 21 (33.3) |
| History of cancer | 42 (13.1) | 30 (11.7) | 12 (19.0) |
| Others | 16 (5) | 12 (4.7) | 4 (6.3) |
| Healthcare workers | 2 (0.6) | 1 (0.4) | 1 (1.6) |

* Pregnancy, obesity (body mass index>30 kg/m^2^), 65-year old subjects and over, nursing home residency regardless of age, type 1 and 2 diabetes, chronic respiratory disease (chronic broncho-pulmonary diseases including asthma, broncho-pulmonary dysplasia and cystic fibrosis, chronic respiratory insufficiency), cardiac disease (congenital heart disease, heart failure, valvular disease, severe arrhythmia, coronary disease), neurological or muscle disease (stroke, severe forms of neurological and muscular disorders, para and tetraplegia with diaphragmatic involvement), renal disease (severe chronic renal insufficiency, nephrotic syndrome), immunosuppressive status (primary or acquired immune deficiency, except regular treatment with immunoglobulins, HIV infection and AIDS, solid transplantation), and others (liver disease, sickle cell disease, health-care professionals).
